# Supplementary material for: Development of a new methodology for the determination of PET microplastics in sediment, based on microwave-assisted acid digestion
Source: PLoS One. 2024 Dec 17;19(12):e0314520. doi: 10.1371/journal.pone.0314520 (PMC11651601; doi:10.1371/journal.pone.0314520)
Supplement: S1 File — (PDF) [file pone.0314520.s001.pdf]

Nov 21, 2024

Method for the determination of PET Microplastics in Sediment

DOI
dx.doi.org/10.17504/protocols.io.5jyl82b77l2w/v1

WALDO EMERZON QUIROZ VENEGAS[1]

[1]Pontificia Universidad Católica de Valparaíso

Laboratorio de Química A…

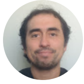

WALDO EMERZON QUIROZ VENEGAS
Pontificia Universidad Católica de Valparaíso

OPEN ACCESS

DOI: dx.doi.org/10.17504/protocols.io.5jyl82b77l2w/v1

Protocol Citation: WALDO EMERZON QUIROZ VENEGAS 2024. Method for the determination of PET Microplastics in Sediment. **protocols.io https://dx.doi.org/10.17504/protocols.io.5jyl82b77l2w/v1**

License: This is an open access protocol distributed under the terms of the **Creative Commons Attribution License**, which permits unrestricted use, distribution, and reproduction in any medium, provided the original author and source are credited

Protocol status: Working
We use this protocol and it's working

Created: July 08, 2024

Last Modified: November 21, 2024

Protocol Integer ID: 103028

Keywords: microplastics, pet, fluorescence, sediment

Funders Acknowledgement:
ANID-FONDECYT
Grant ID: 1230585

Abstract

The methodology is based on a direct acid attack of HNO_3/HCl 3:1 from the wet sediment sample, assisted by microwaves digestion system. No drying, sieving or flotation procedures are neccesary. As a detection method we use induced fluorescence with a UV camera, Red Nile as a fluorophore and a cell phone camera.

Attachments

DOCX

S1.docx

412KB

Guidelines

no comments

Materials

The reagents and solvents used were Scharlau HPLC grade acetone, Supelco suprapure hydrochloric acid, Supelco suprapure nitric acid (65%), Milli-Q laboratory water, and Nile Red Sigma Aldrich.

Analytical-grade chitin and purified marine sand (both from Sigma Aldrich) and crude cellulose were used to simulate the sedimentary matrix.

The MPs used in the experiments represent the polymers most found in the marine environment: polyethylene terephthalate (PET), low-density polyethylene (LDPE), polystyrene (PS) and polypropylene (PP). MPs were made from virgin plastic resins of each type of polymer. A grinding system was implemented to ensure that the resin is free from contact with any other polymer. The grinding process

involves chopping at 5-second intervals with 10-second breaks to prevent heating and possible structural changes in each polymer. Subsequently, the material is screened through 1.0, 0.5 and 0.15 mm mesh, resulting in two working fractions (1-0.5 mm and <0.5-0.15 mm), which are stored in stainless steel containers.

Safety warnings

no comments

Ethics statement

no comments

Before start

Determination of PET in sediments

New methodology for the determination of PET microplastics in sediment, based on microwave-assisted acid digestion

1 Sample Digestion:

1. Weigh 1g of wet sediment and place it in the microwave digestion vial.
2. Add 20 ml of a 3:1 v/v HNO_3/HCl mixture to the sample in the microwave digestion vial. Seal and place it in the microwave equipment.
3. Digest the sample for 1 hour at 60ºC.
4. Filter the digestion solution through a 0.45 µm membrane filter. Quantitatively rinse all material from the digestion vial with deionized water using a wash bottle. Before finishing the filtration, rinse thoroughly with water to dislodge any digested material clinging to the filtration vessel walls. Also, use circular motions with deionized water from a wash bottle to ensure that the undigested solids are centered on the filtration membrane.
5. Carefully transfer the membrane to a 100 mm diameter Petri dish. Cover the Petri dish with aluminium foil and make small holes in the top.
6. Place the dish in a drying oven or incubator at 60ºC for one hour. After this time, let the dish cool and proceed to the staining stage.

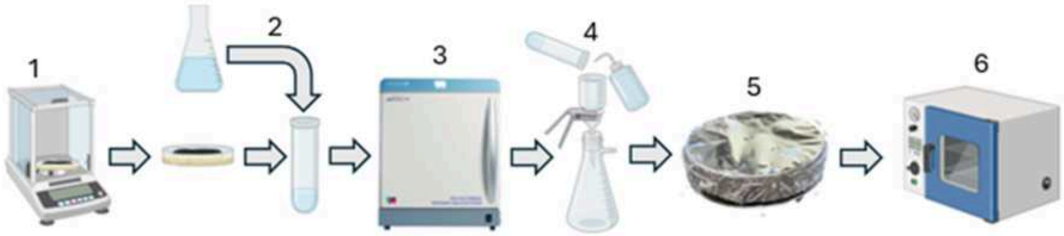

Sample digestion

Staining step:

1. Prepare a Red Nile (RN) solution at a concentration of 10 mg L^{-1} at 1% v/v in 99.5% v/v acetone.
2. Add 20 ml of the RN solution to the Petri dishes containing the filtered solid and membrane. Cover the dish with its corresponding glass

lid.
3. Place the Petri dish in the incubator for 10 minutes at 50ºC.
4. After the incubation time, quickly cool the Petri dish in an ice water bath. Let it stand for 20 minutes.
5. Filter the solution with abundant deionized water through a 0.45 µm membrane filter.
6. Place the membrane with the stained material back into the same Petri dish. Cover with aluminum foil and make holes.
7. Place in the incubator and dry at 60ºC for one hour. After this time, let the dish cool and proceed to the stage of taking photographs and counting particles.

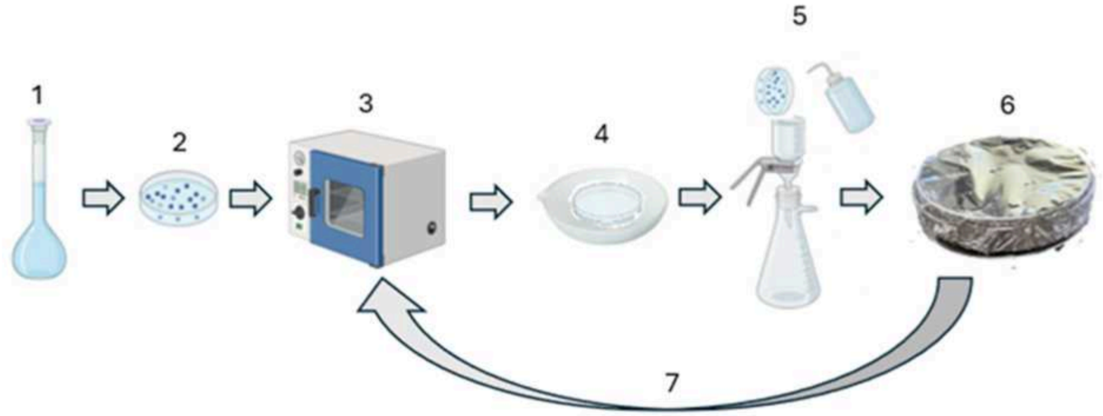

Staining step

Photograph Taking and Particle Counting:

1. Uncover the dish and carefully transfer the filters to the glass lid of the Petri dish. The dish containing the solid material should be moved horizontally until a uniform monolayer is obtained across the surface.
2. Insert each dish separately into the UV chamber, verify that the excitation energy is at 254 nm, and take the corresponding photograph.
3. For particle counting, load the photograph into the ImageJ software.
4. Identify PET particles by their characteristic color and manually count them in the software as follows:

  - Select the multipoint function from the toolbar.
  - Position the cursor over the particle and right-click. As selections increase, the software will perform the count, and each selected particle will be identified with its respective number.

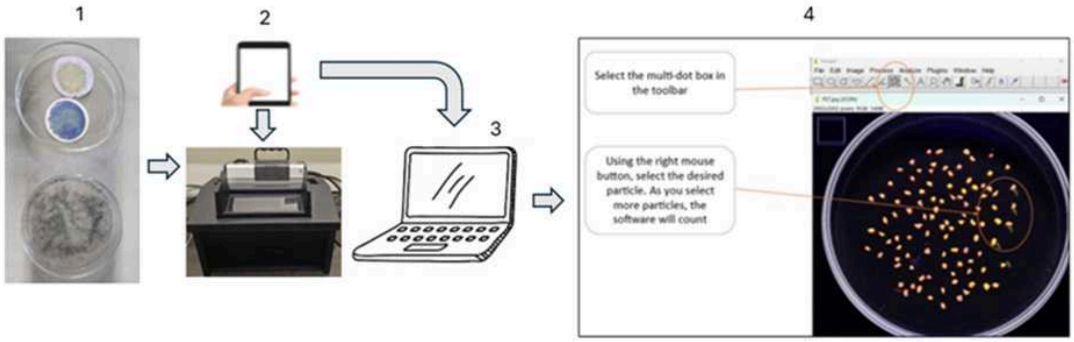

Particle Counting

Protocol references

no comments
