# Supplementary material for: Development of a new methodology for the determination of PET microplastics in sediment, based on microwave-assisted acid digestion
Source: PLoS One. 2024 Dec 17;19(12):e0314520. doi: 10.1371/journal.pone.0314520 (PMC11651601; doi:10.1371/journal.pone.0314520)

S4

Image behind Table 1 values

Recovery image using NaCl flotation solution


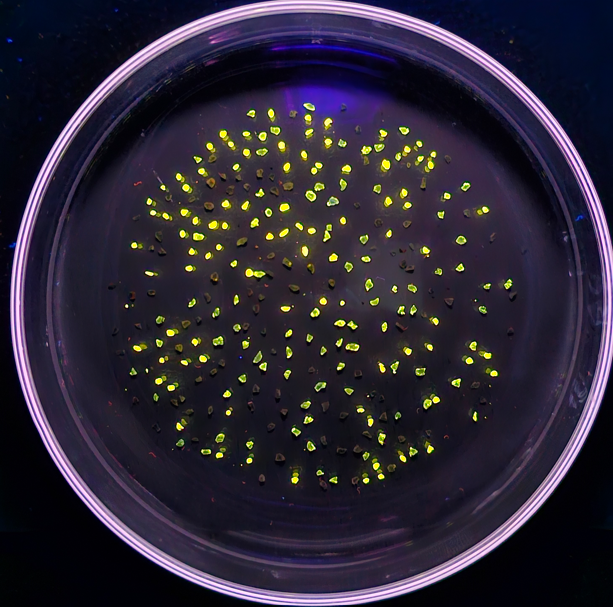

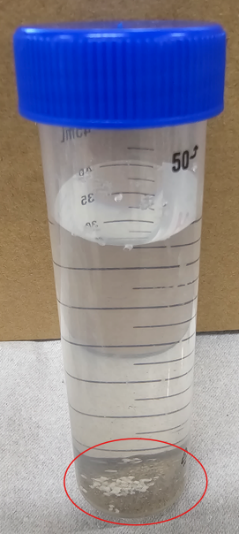


Recovery image using CaCl2 flotation solution


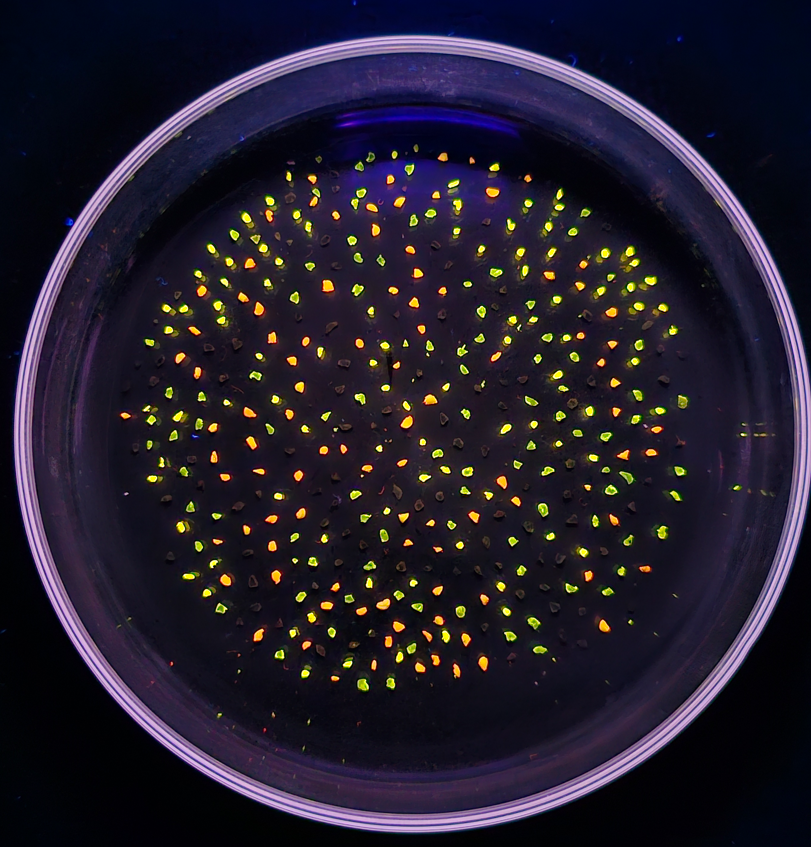

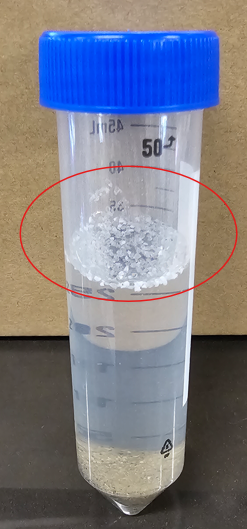

Supplement: S1 Fig — (DOCX) [file pone.0314520.s008.docx]
