# Supplementary material for: Development of a new methodology for the determination of PET microplastics in sediment, based on microwave-assisted acid digestion
Source: PLoS One. 2024 Dec 17;19(12):e0314520. doi: 10.1371/journal.pone.0314520 (PMC11651601; doi:10.1371/journal.pone.0314520)

S6

Figure 8. Image used for manual PET MPs quantification. Spike and recovery of PET microplastics (MPs) was conducted under the following digestion conditions: isolated PET microplastic particles (MPs PET), PET microplastics spiked into marine sediments (MPs PET with sediments), and a mixture of microplastic polymers (MPs Mix).

MPs PET


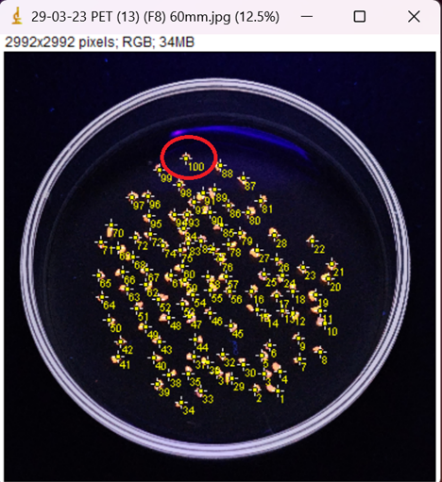


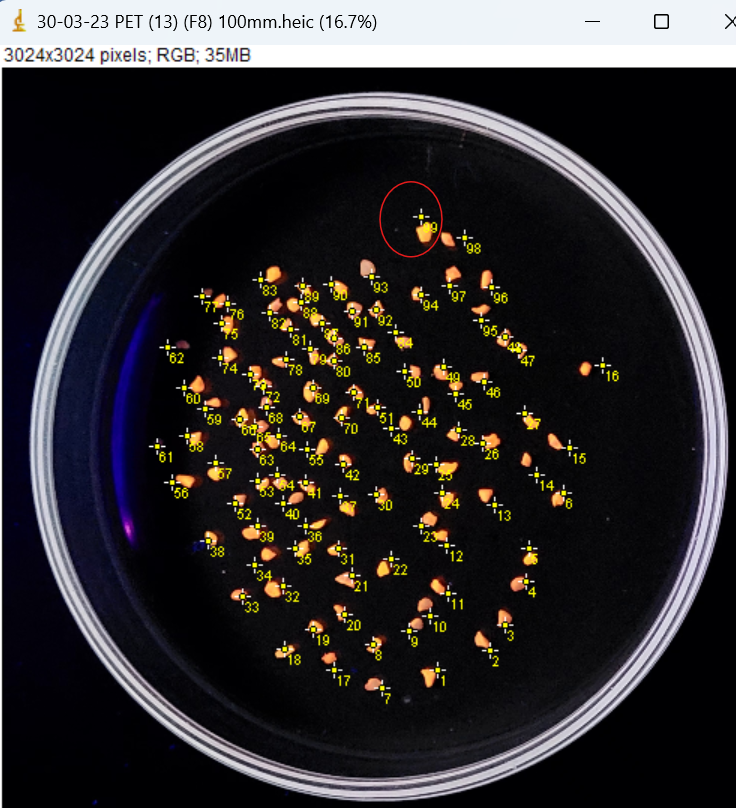


PET MPs spiked ins sediment


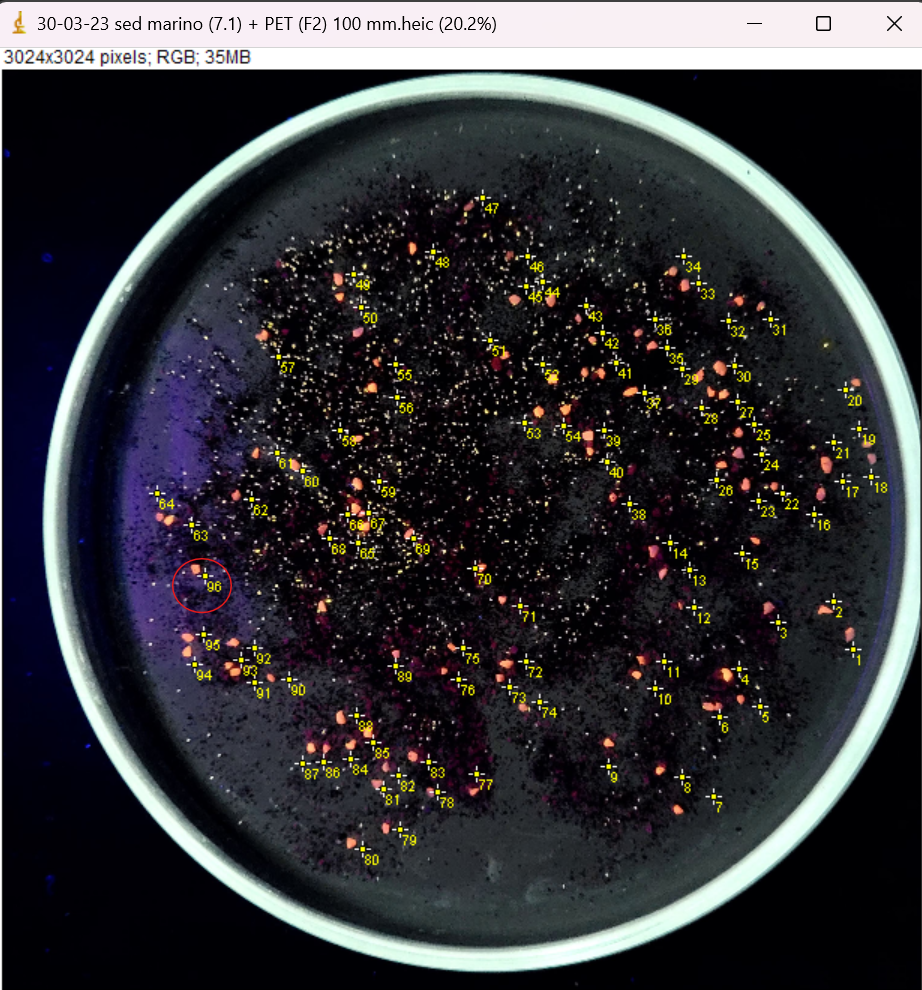

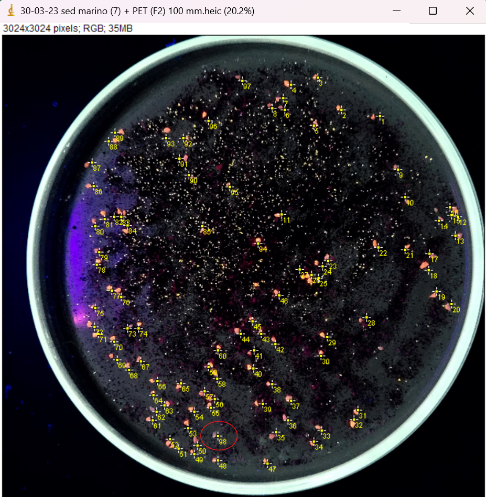


PET MPs with polymer mix


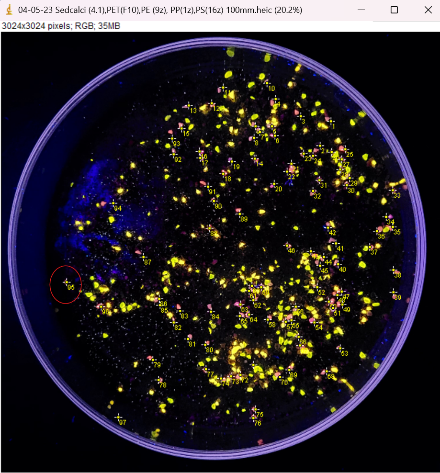

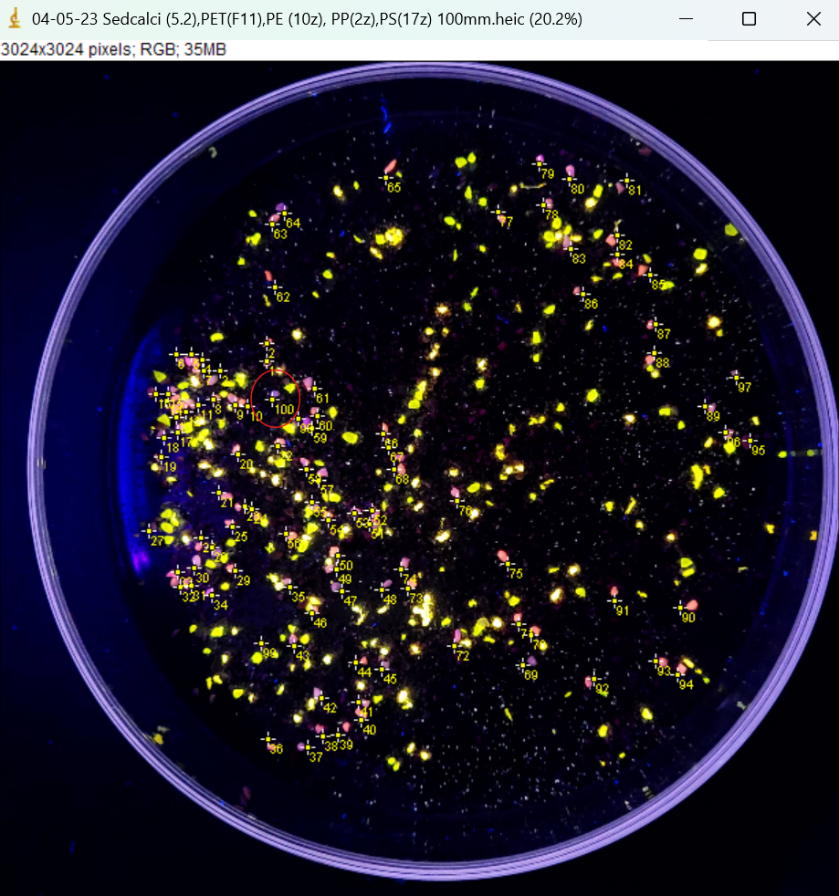

Supplement: S2 Fig — Spike and recovery of PET microplastics (MPs) was conducted under the following digestion conditions: isolated PET microplastic particles (MPs PET), PET microplastics spiked into marine sediments (MPs PET with sediments), and a mixture of microplastic polymers (MPs Mix). (DOCX) [file pone.0314520.s009.docx]
