# Supplementary material for: Development of a new methodology for the determination of PET microplastics in sediment, based on microwave-assisted acid digestion
Source: PLoS One. 2024 Dec 17;19(12):e0314520. doi: 10.1371/journal.pone.0314520 (PMC11651601; doi:10.1371/journal.pone.0314520)

S9

Image used for manual identification of PETs MPs in Figure 12.

**Images**

TAIRA


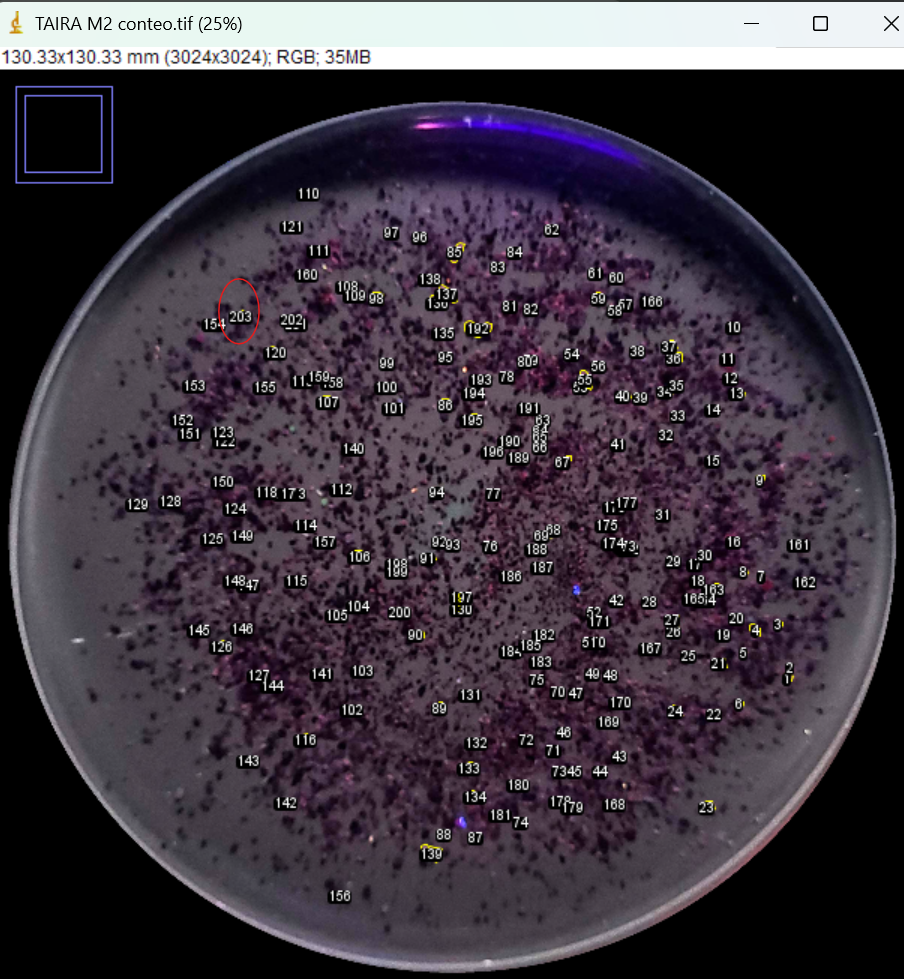

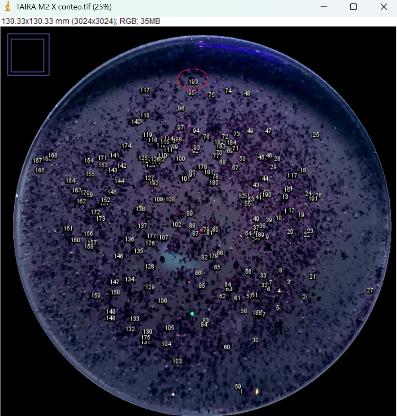

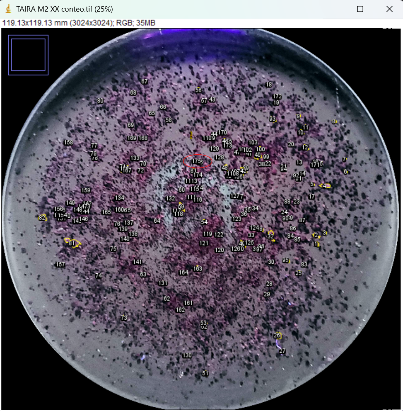


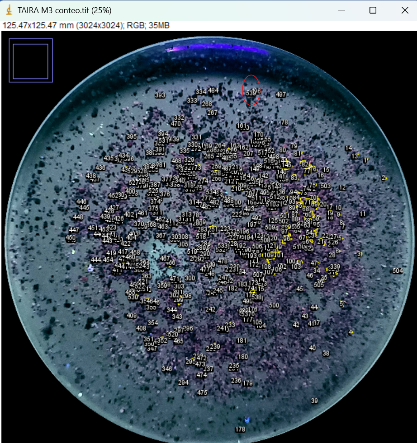

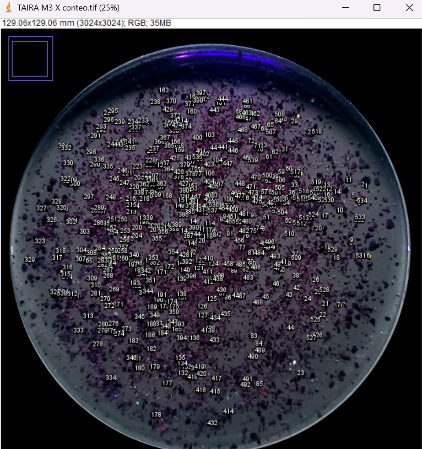


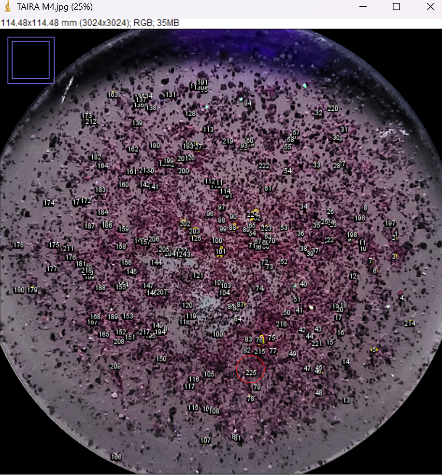

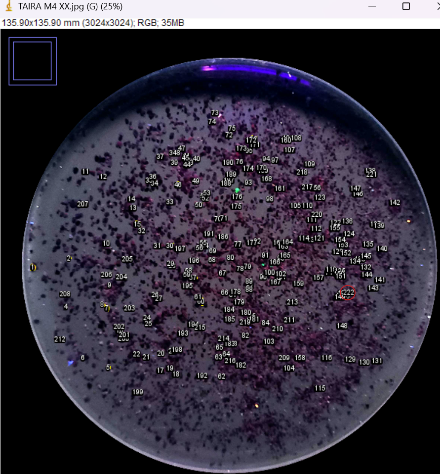

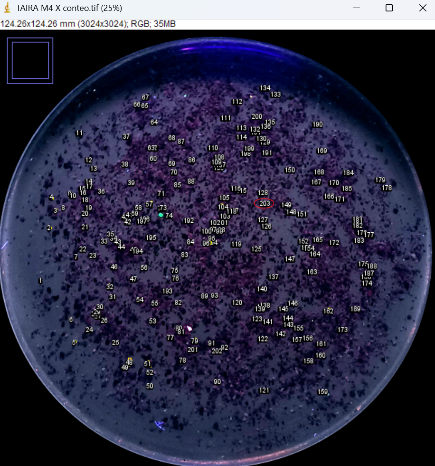


CHIUCHIU


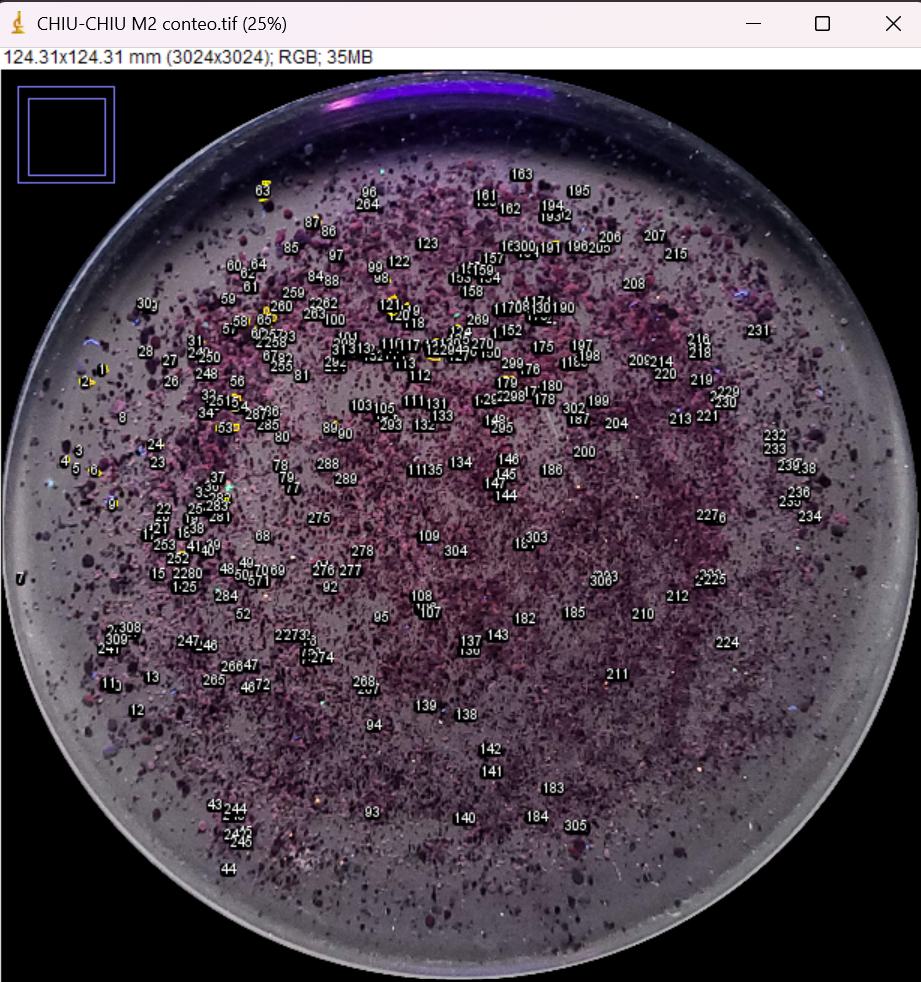

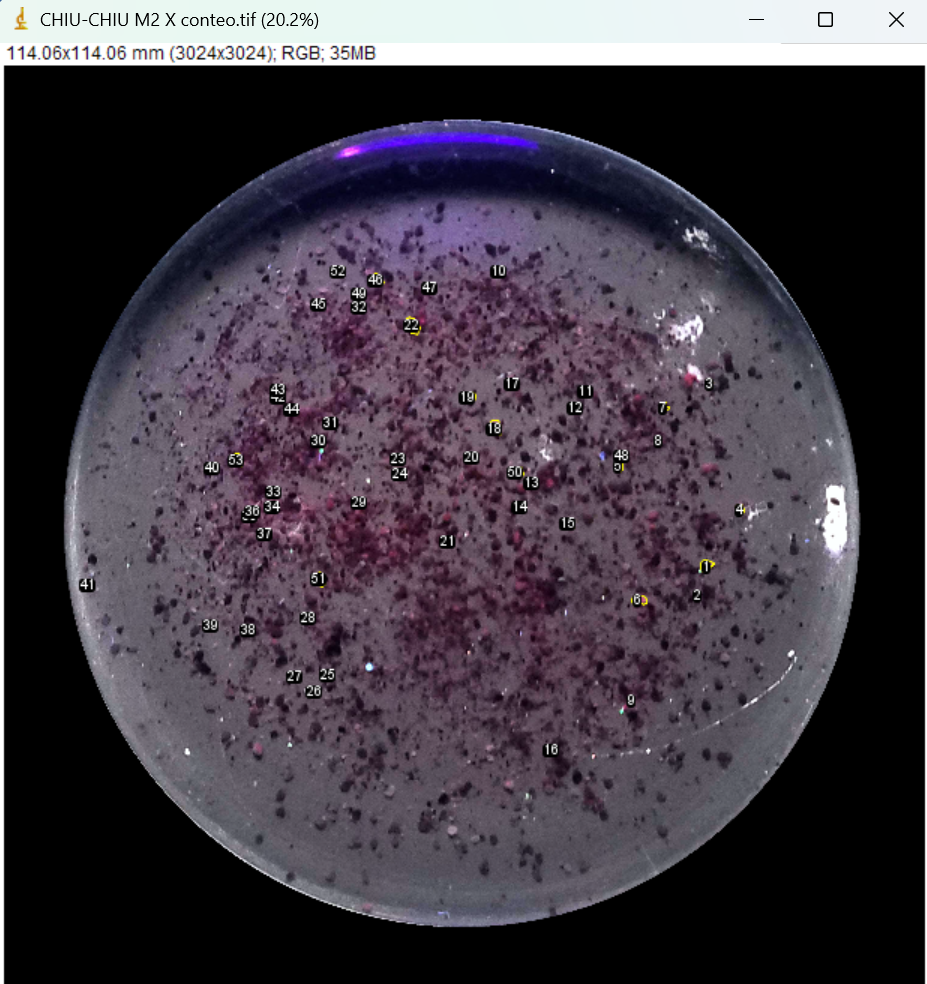

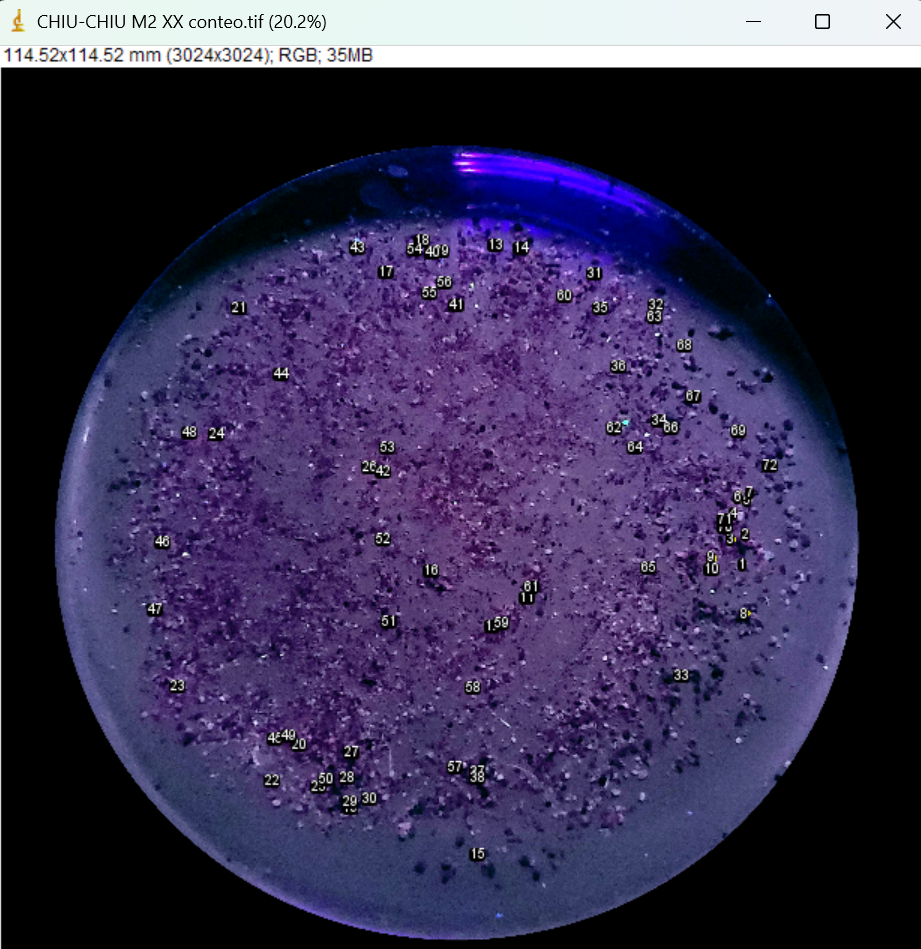


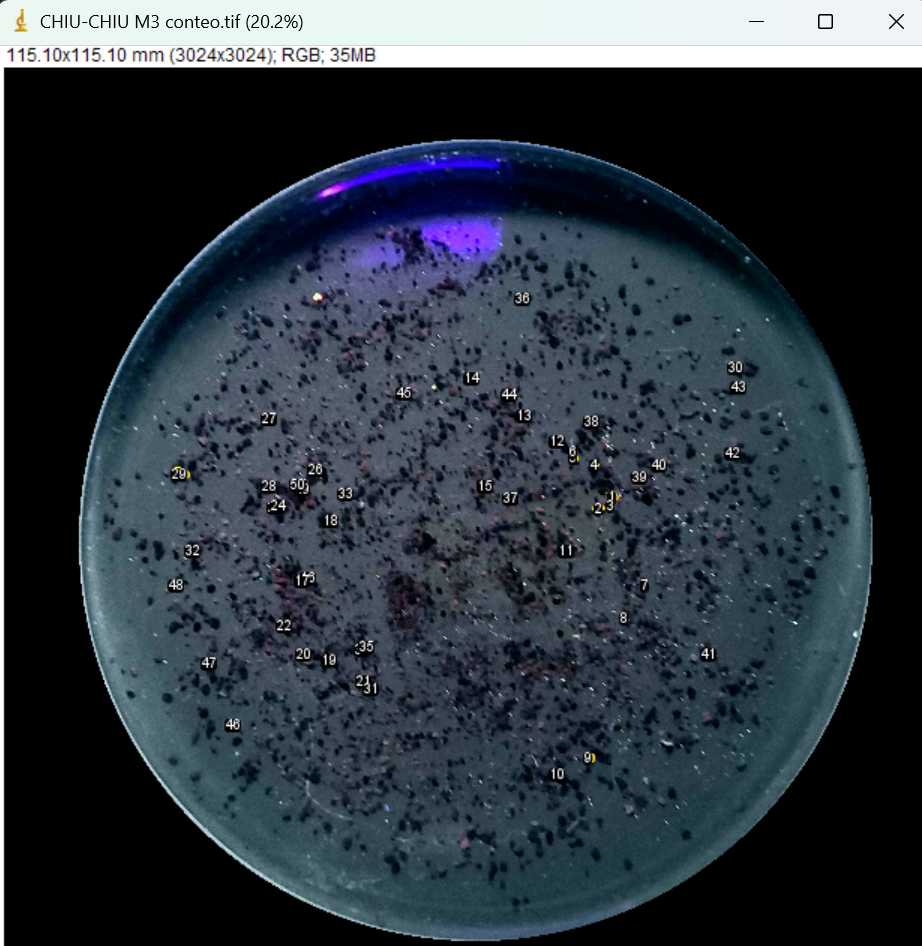

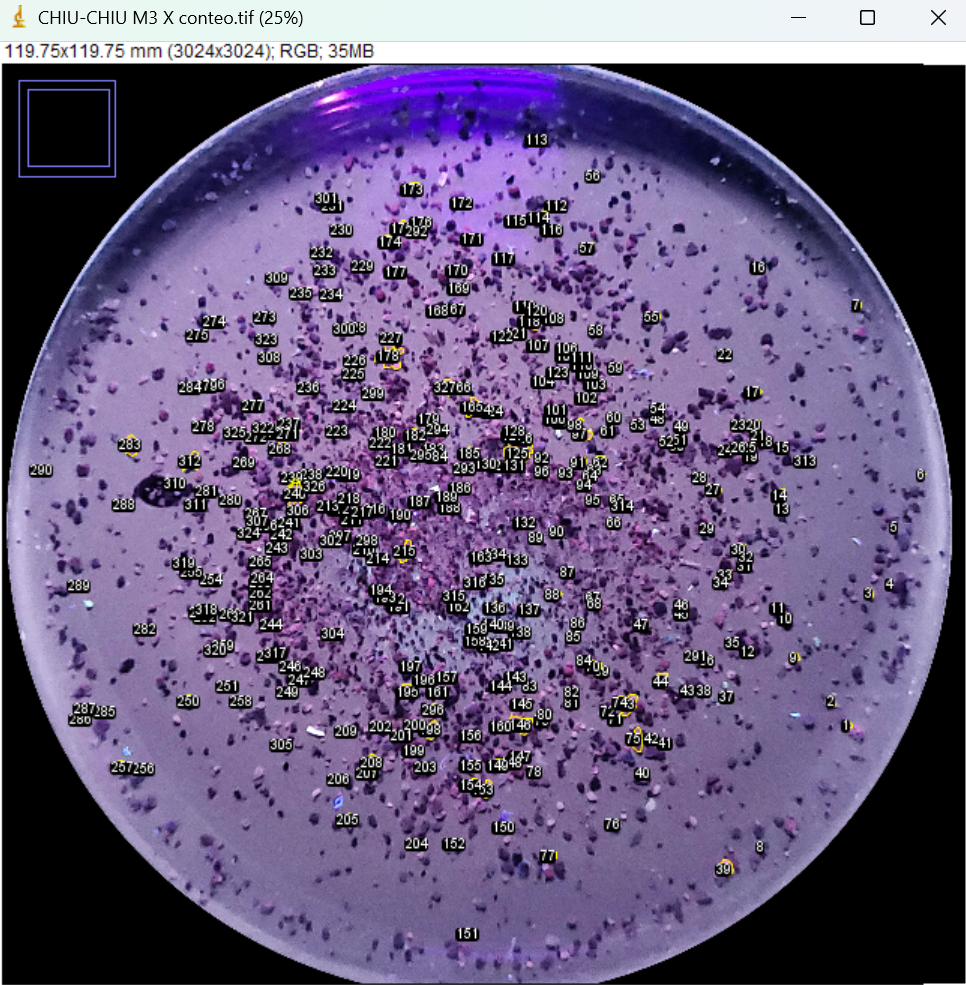

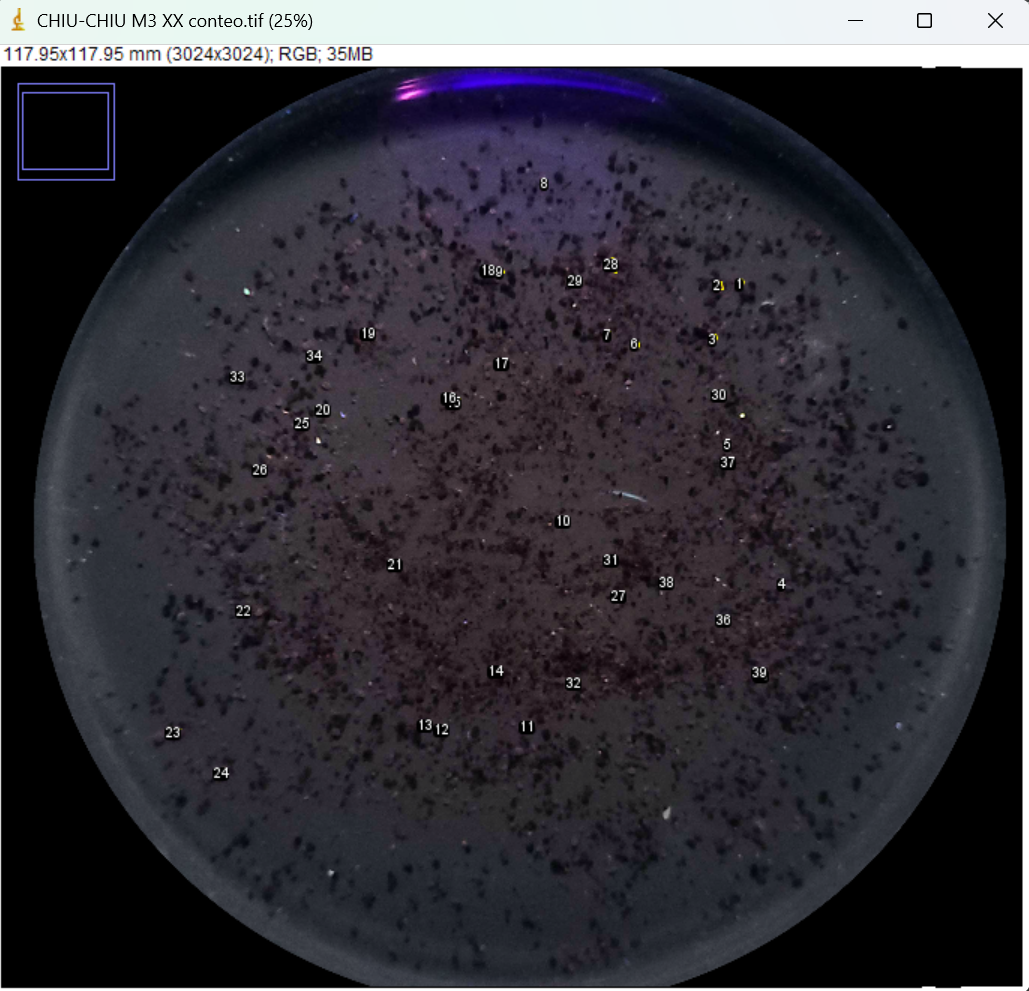


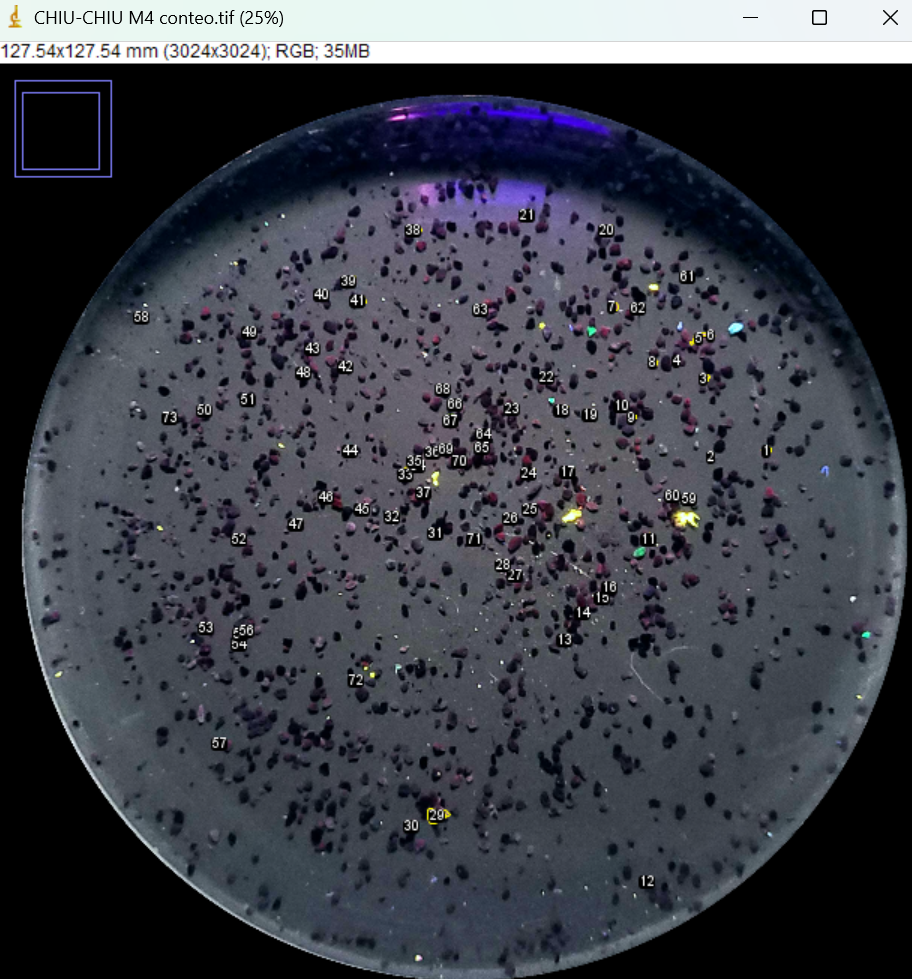

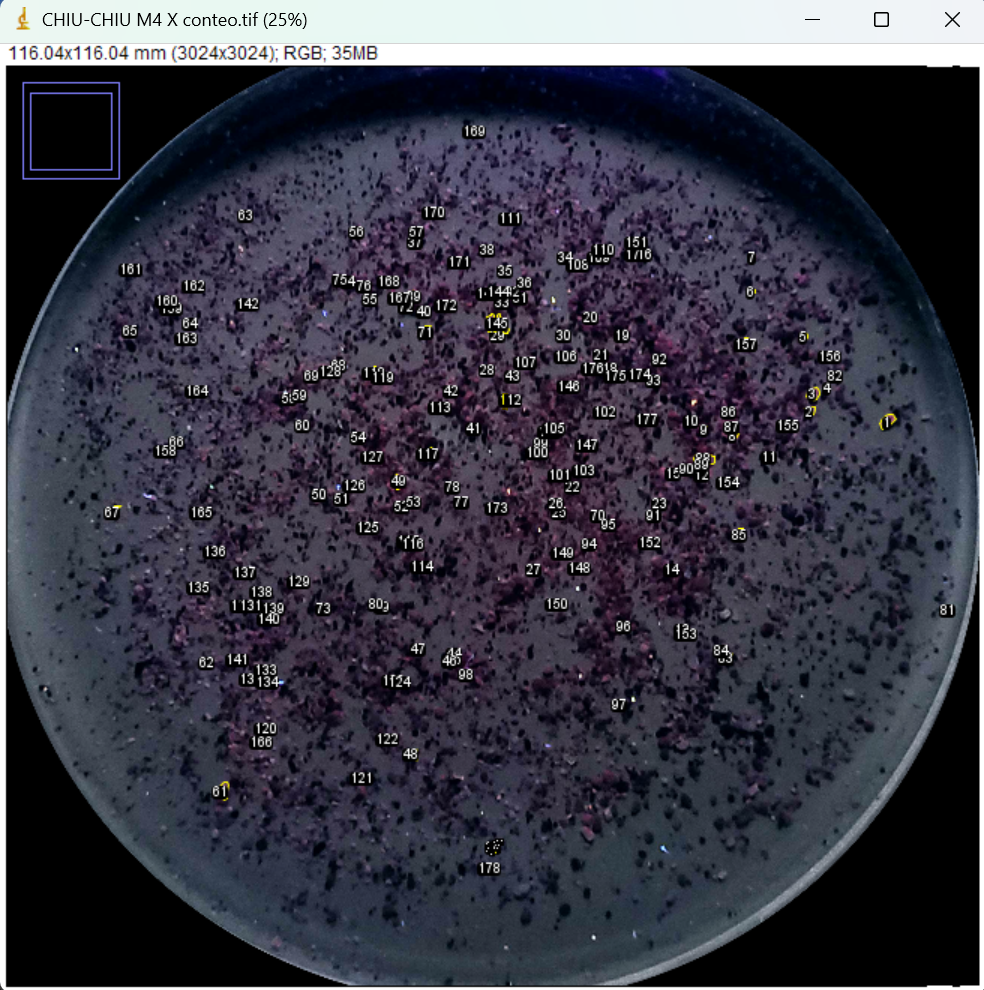

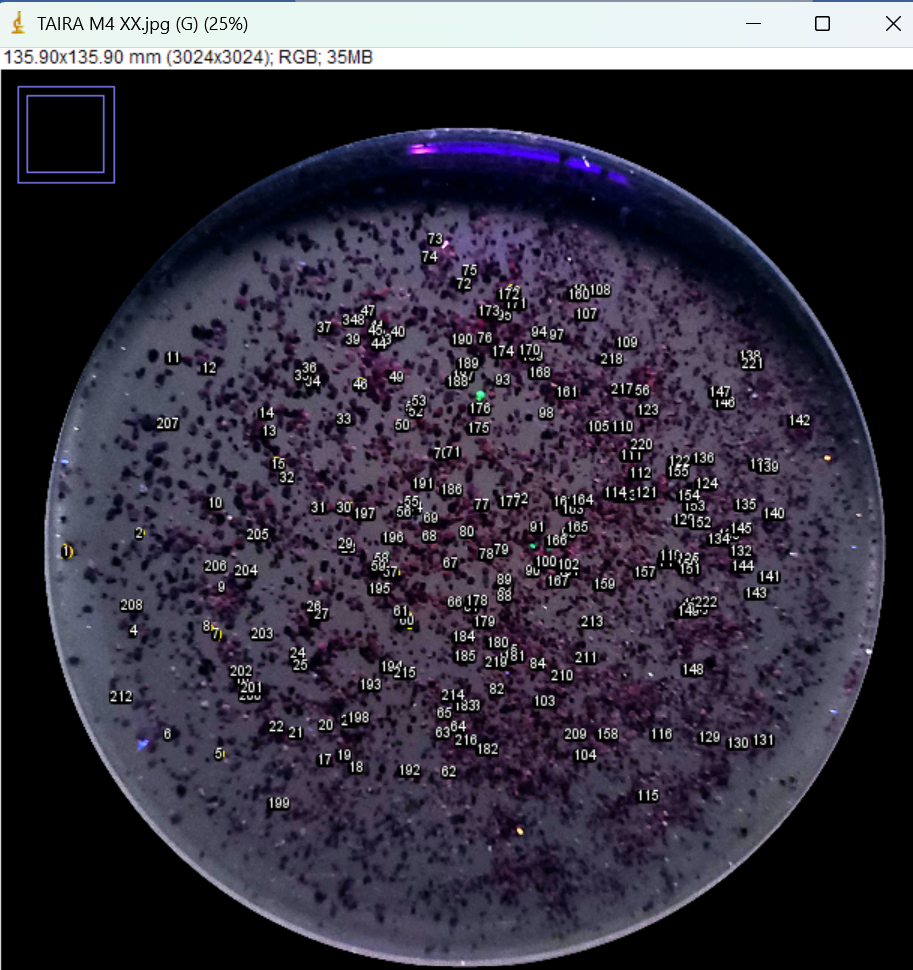


DESEMBOCADURA (Mouth River)


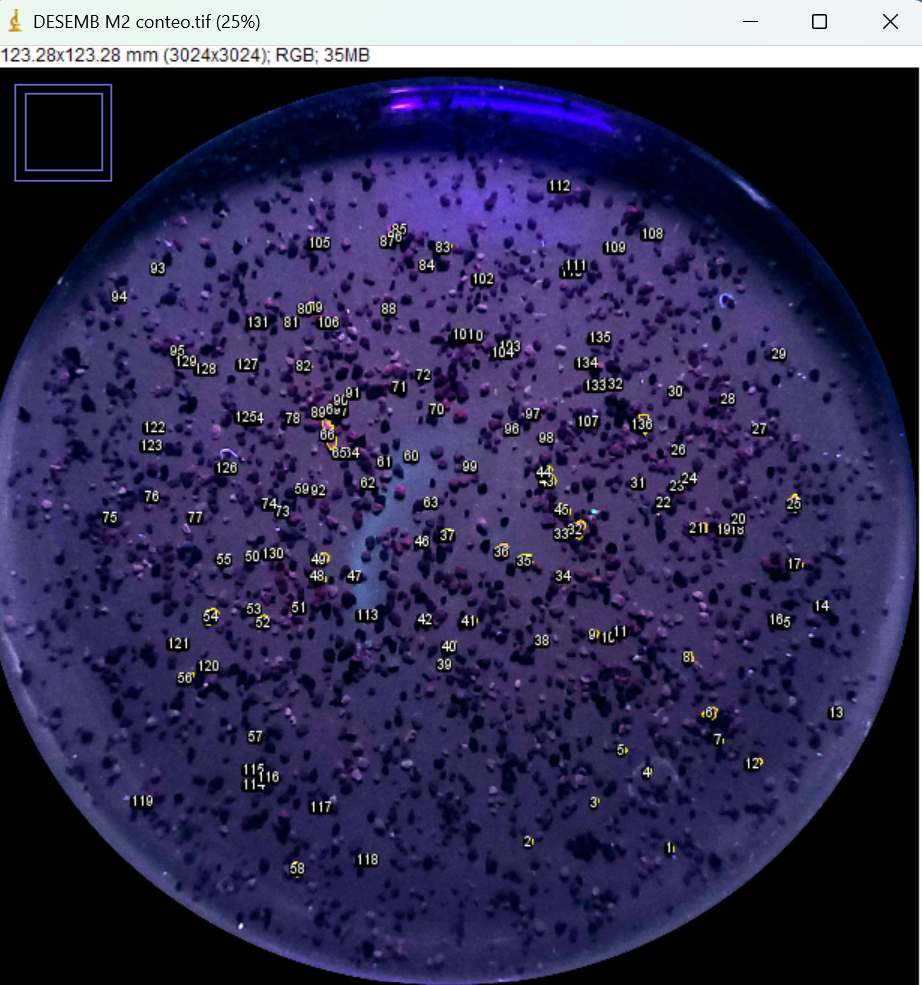

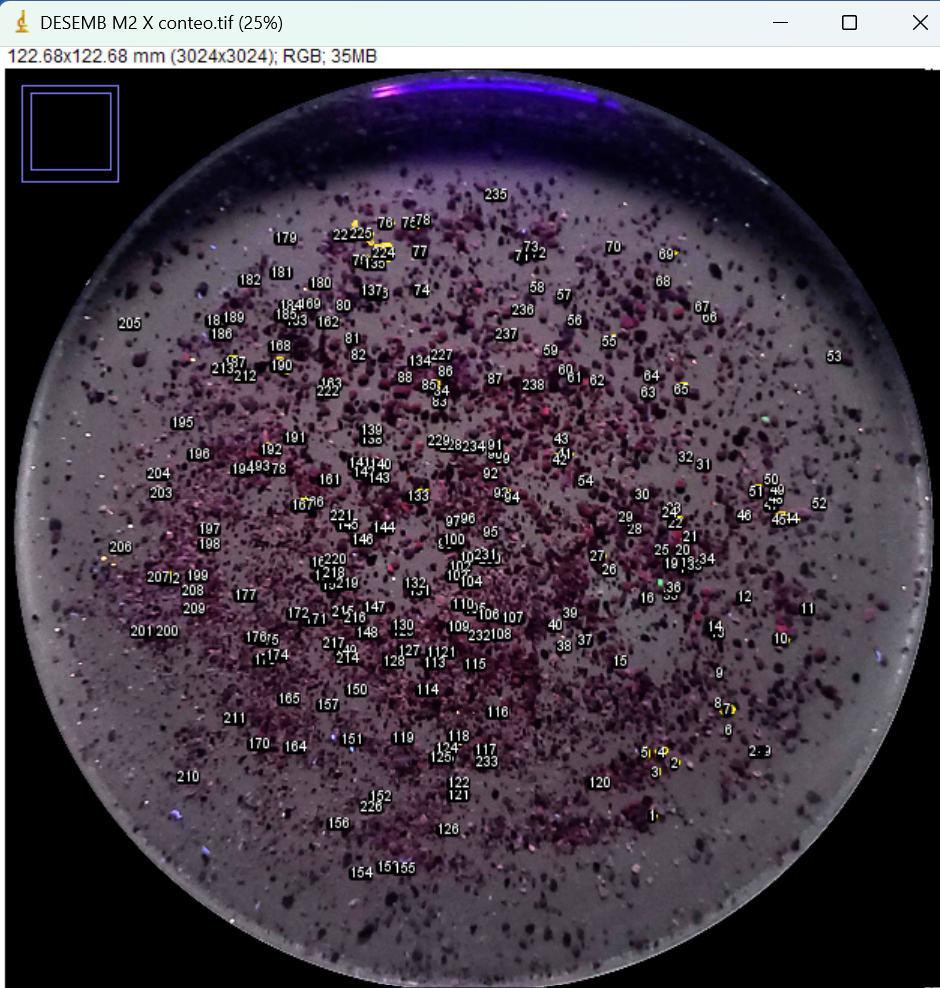

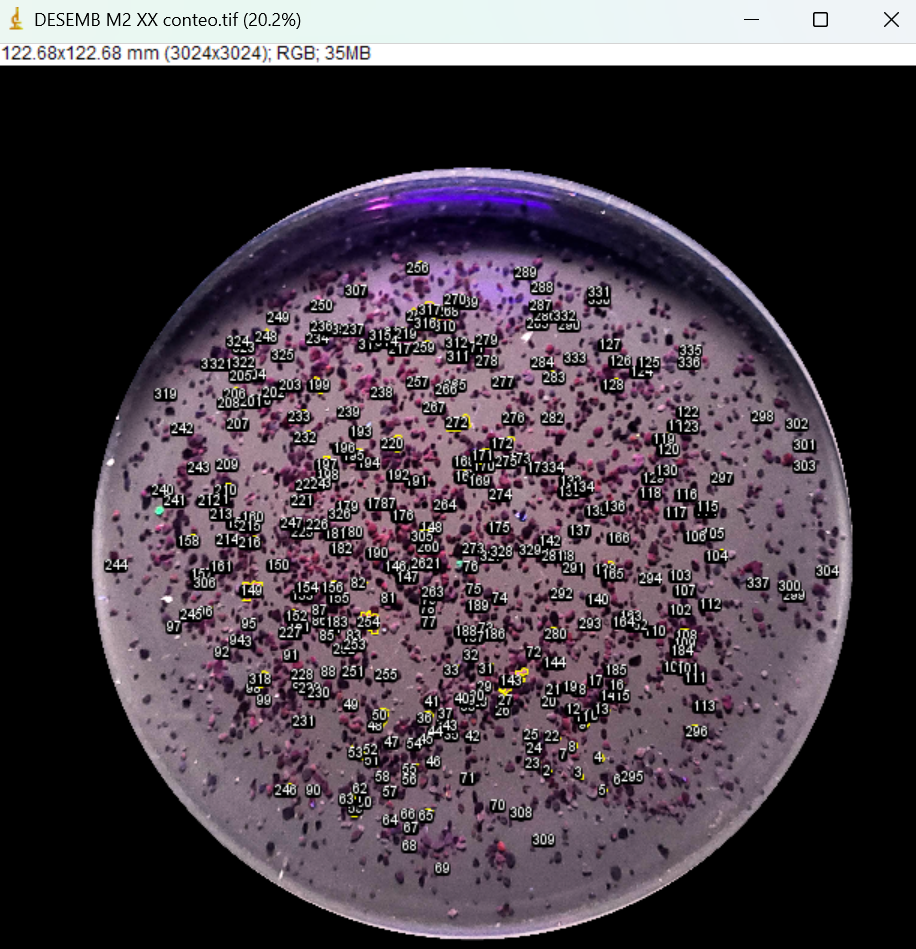


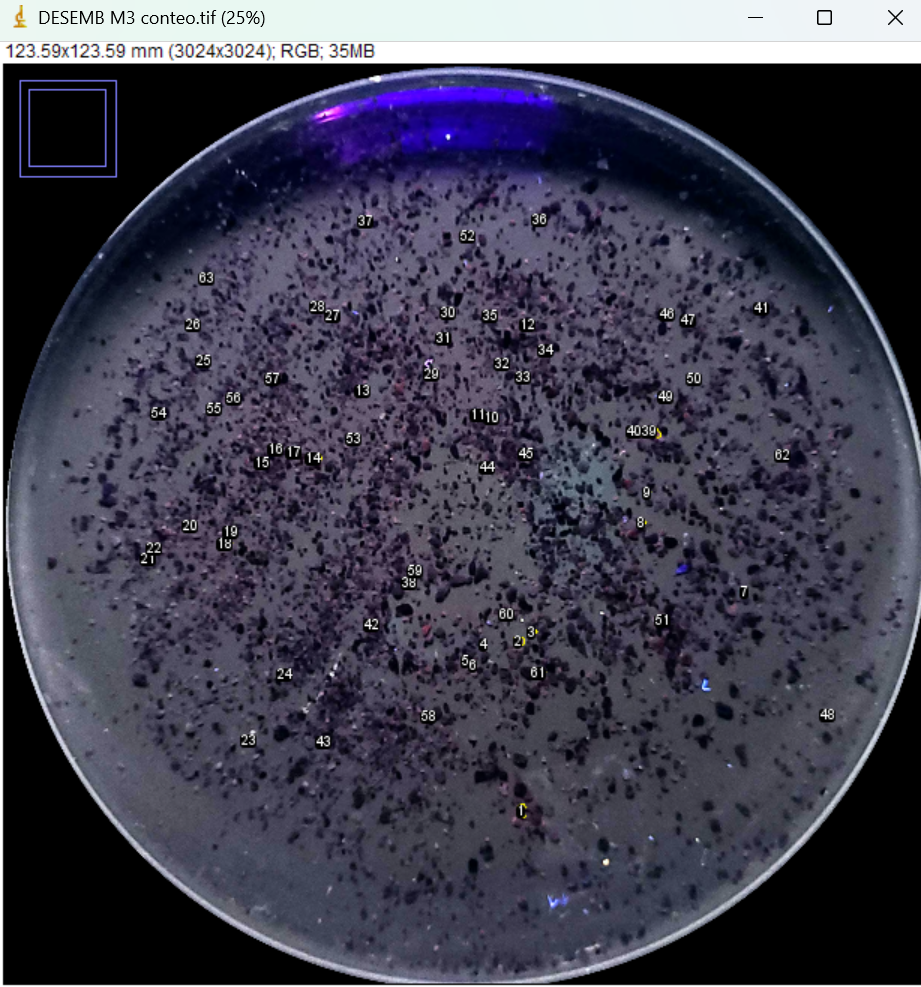

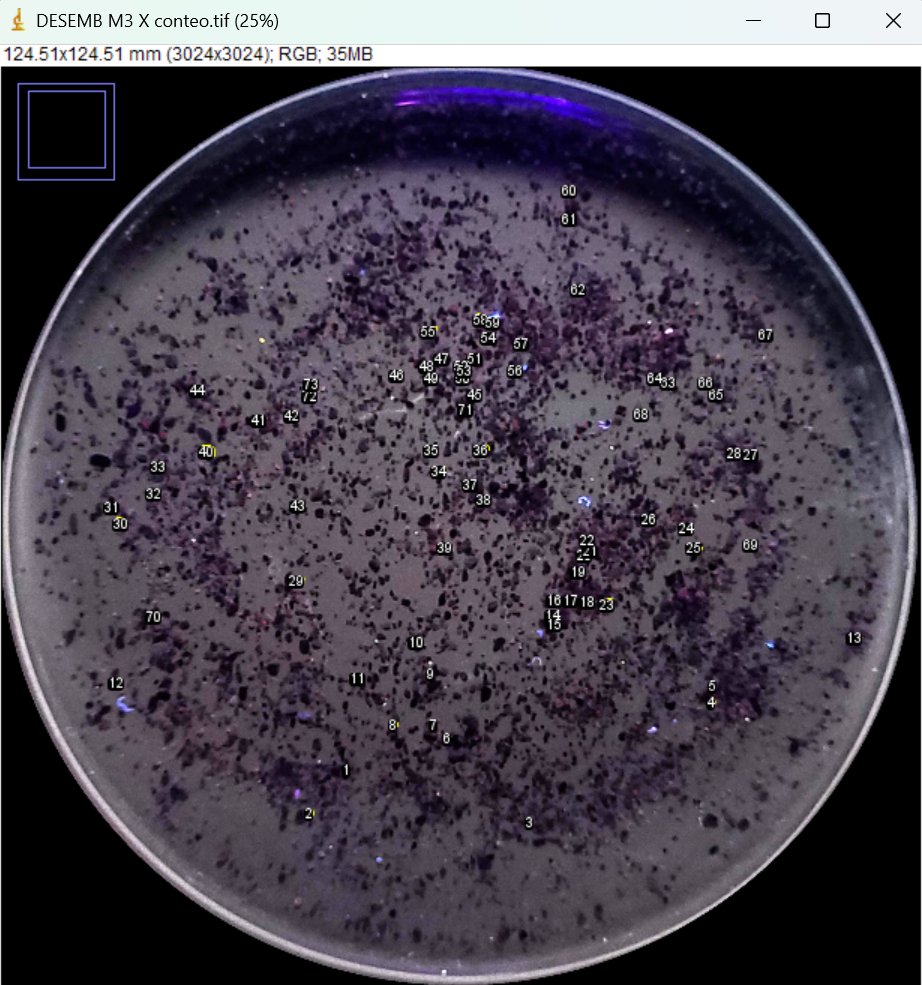

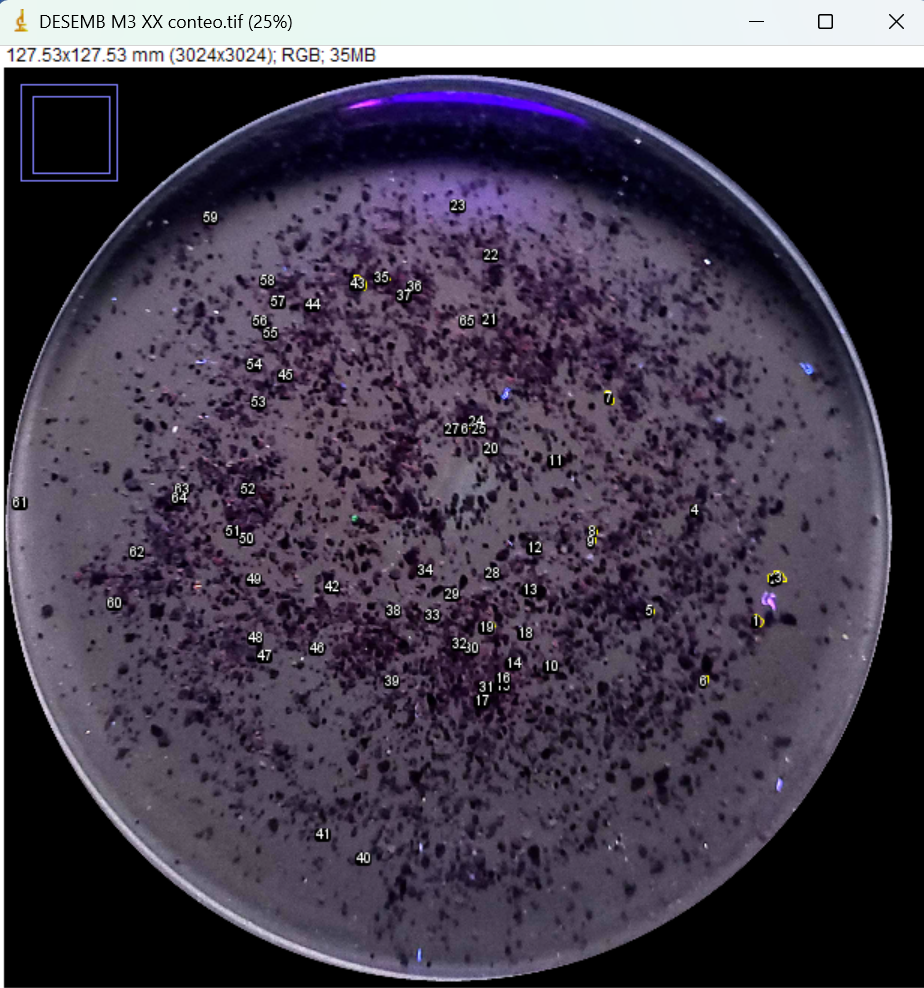


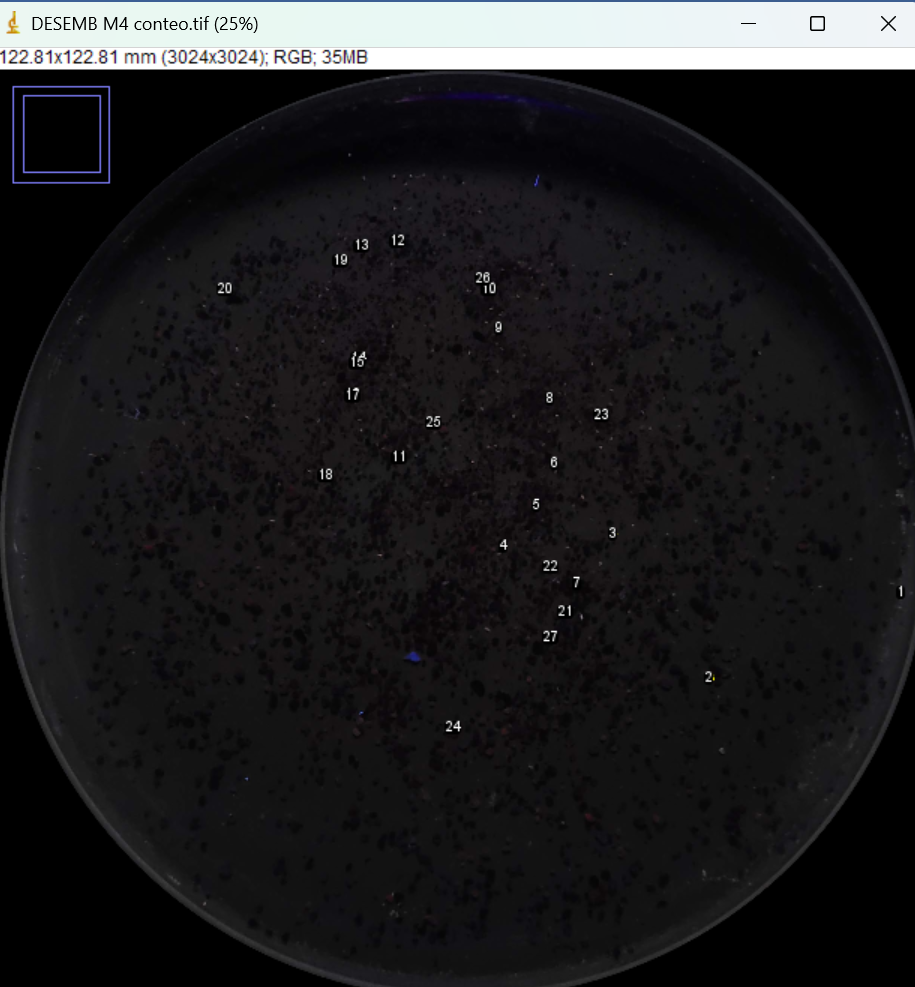

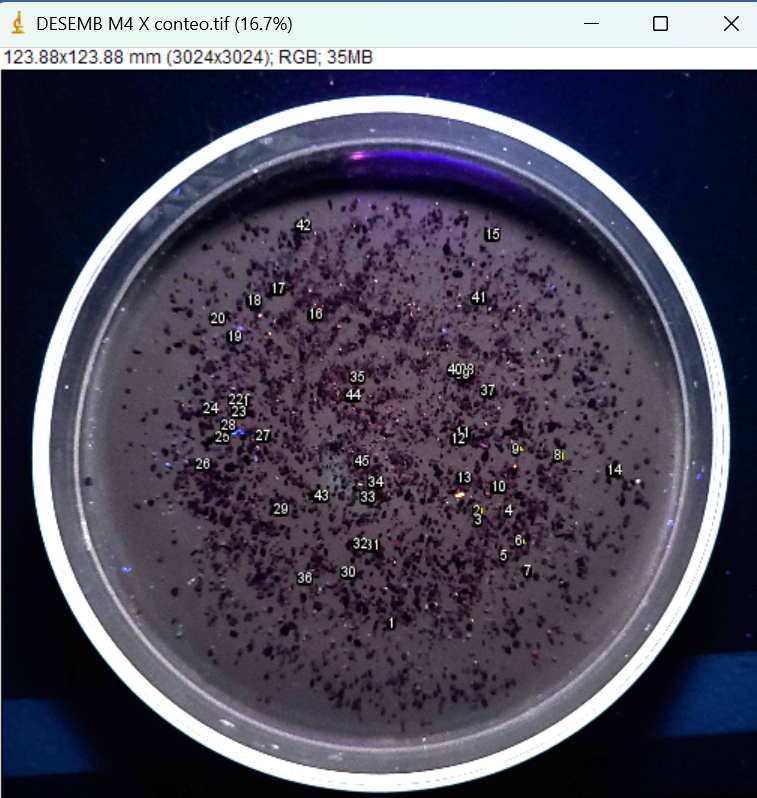

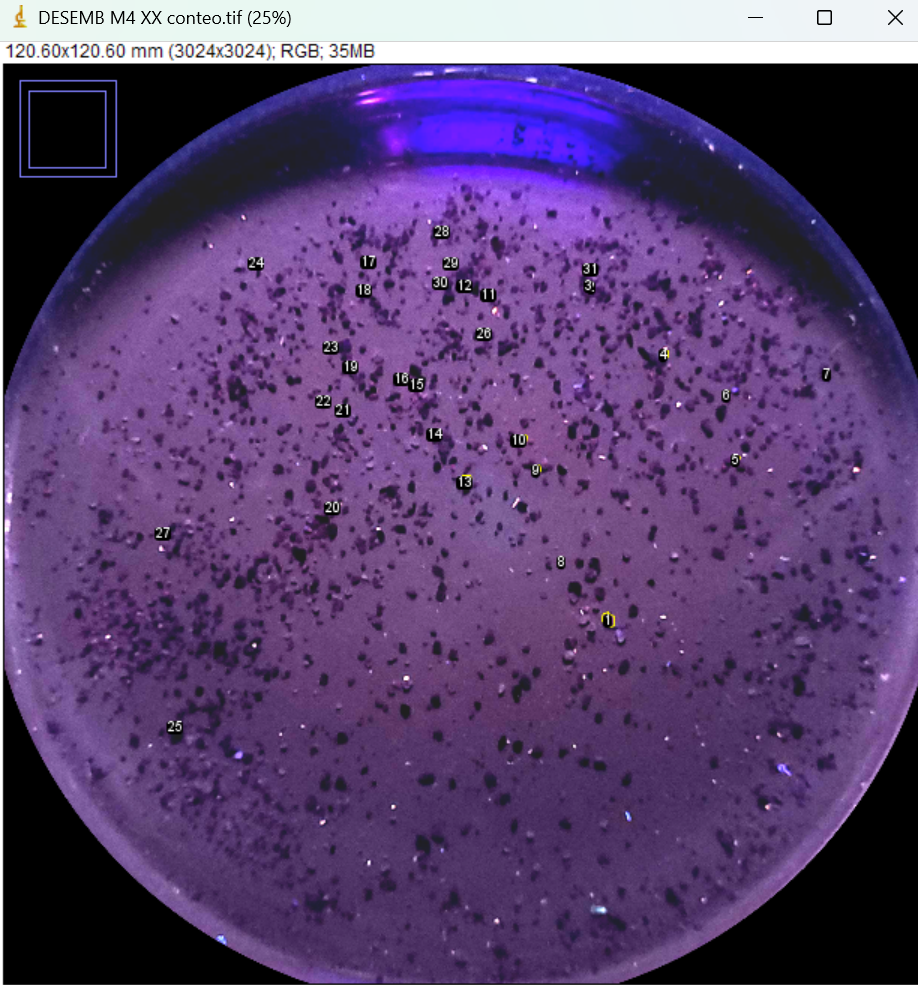


QUILLAGUA


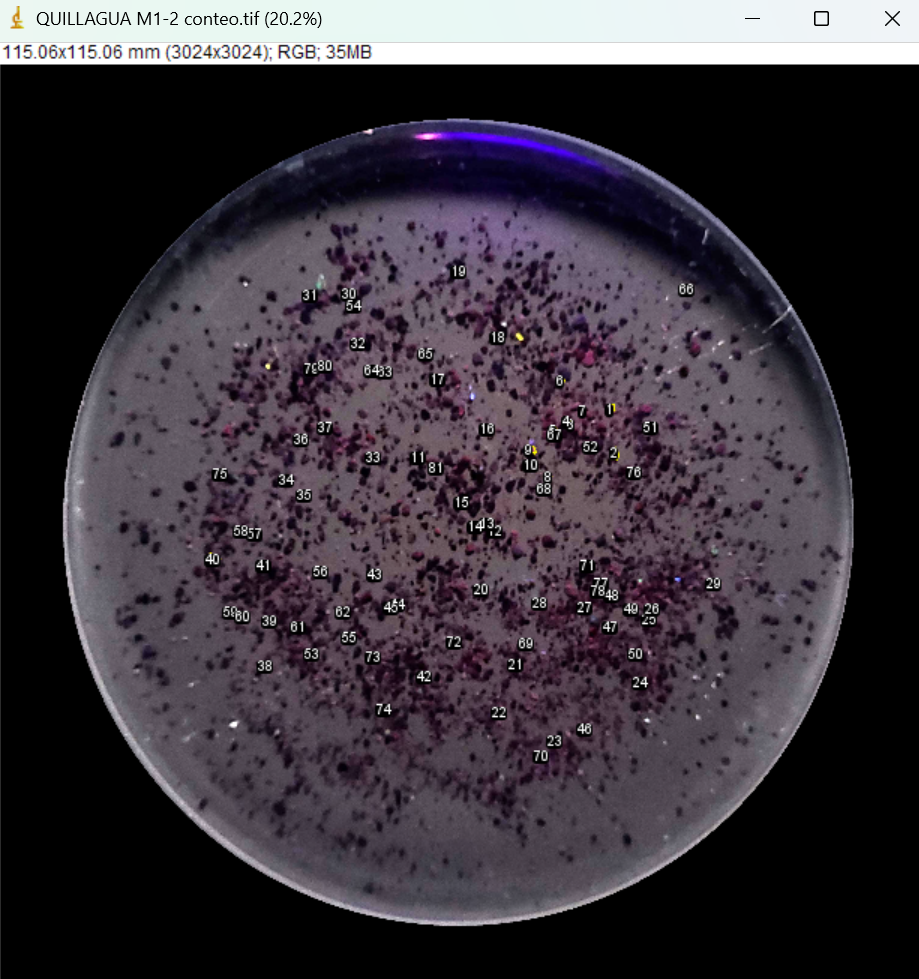

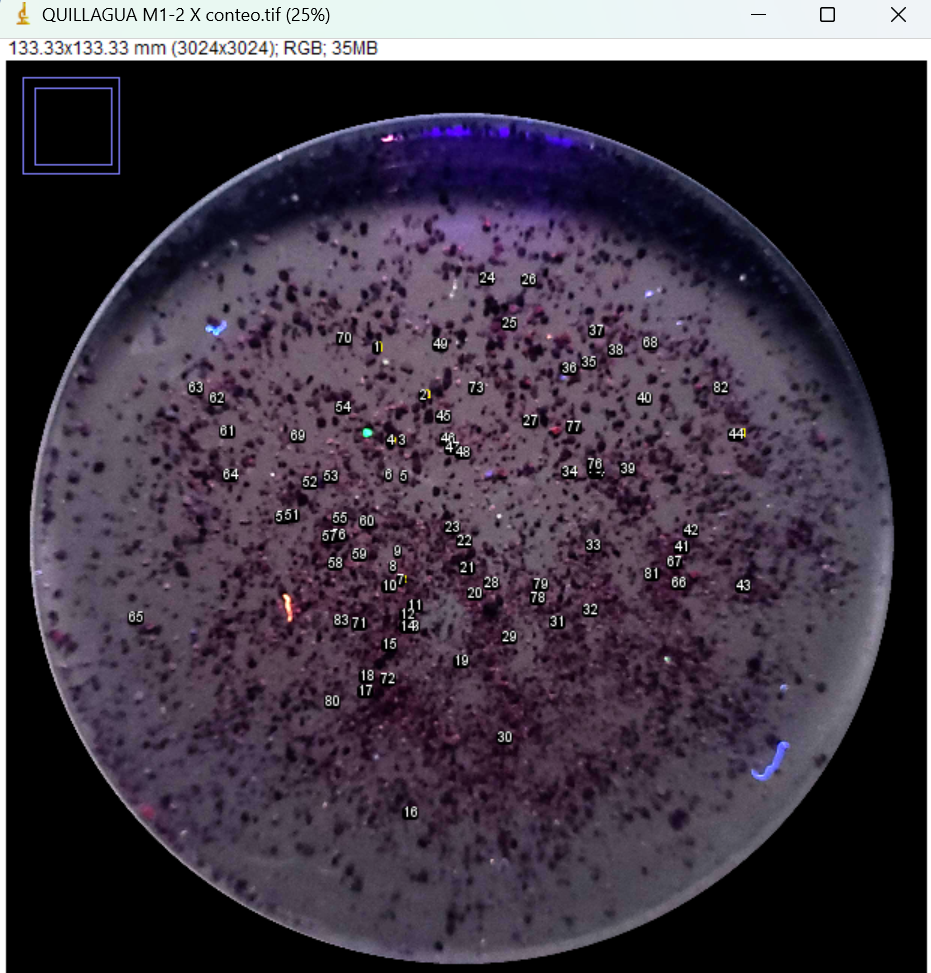

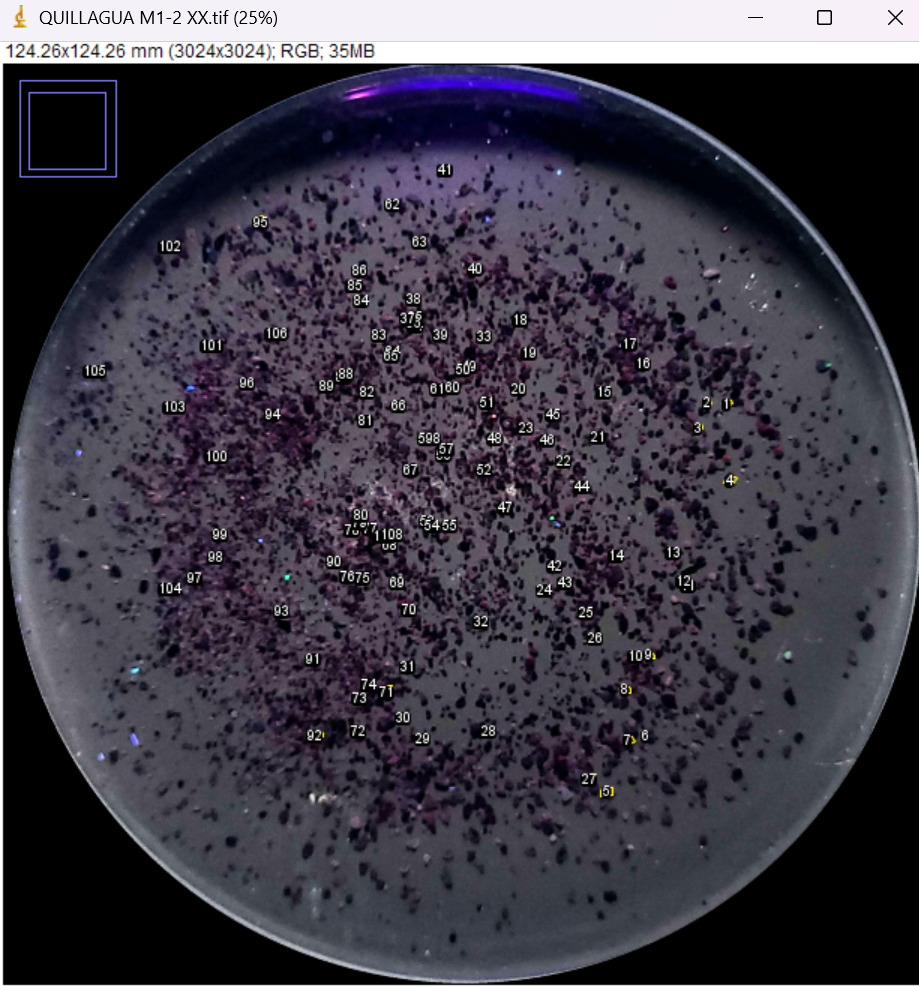

Supplement: S3 Fig — (DOCX) [file pone.0314520.s010.docx]
