# Supplementary material for: Development of a new methodology for the determination of PET microplastics in sediment, based on microwave-assisted acid digestion
Source: PLoS One. 2024 Dec 17;19(12):e0314520. doi: 10.1371/journal.pone.0314520 (PMC11651601; doi:10.1371/journal.pone.0314520)
Supplement: Attachment — Submitted filename: Review comments.pdf [file pone.0314520.s011.pdf]

The authors have formulated a way to recover PET particles from the sample by subjecting the samples to differential conditions, including different temperature and excitation wavelength. These conditions have successfully eliminated other interferences at each consequent stages and resulted in the detection of PET MPs. I would like to suggest the following:

General comments: Applies to the entire manuscript.

1. Check the manuscript for grammatical and spelling errors. For example: In keywords “Red Nyle” is not correct.
2. For all the statistical representation, mention the software used for the preparation of graphs. Additionally, include a statistical analysis section for this in the methodology head.
3. I suggest it would be appropriate if a pictorial representation or a flowchart is included for methodology, including the effect of each step (such as elimination of chitin fluorescence after digestion)
4. The font consistency is lacking. For example: “Result and Discussions” and “Conclusions” are in different forms; also check font in Table 1.

Hence, I recommend the authors to rewrite the manuscript considering all the above stated suggestions and thus improving the overall flow of the manuscript.

Specific comments:

Major comments-

1. The addition of the possible real-life applications of this methodology would be beneficial for the readers. Additionally, the methodology referred to in reference number 9 has successfully recovered five polymer types. I suggest the authors to clearly include a reason for their focus only on PET.
2. It would be effective if the authors stated the reasons for each and every step as it will provide great understanding for the readers.
3. Line 130- It would be good if the authors explained the use of only 254nm wavelength filter and not the 365nm wavelength as mentioned in the reference paper. Also state the efficiency of this method over already existing methods. Additionally, clarification of the possibility to recover other particles using different excitation wavelengths would be an added advantage. This information would be helpful since many researchers aim to isolate all the potential plastic constituents from the environmental samples. Again,

I suggest the authors to clearly include a reason for their focus only on PET. For this addition of microscopic images depicting the particles in bright field, the selected fluorescence wavelength (254nm) and in other wavelengths, superimposed on each other would substantiate the present study.

Minor comments-

1. Considering that the density of PET is less than that of $CaCl_2$, causing it to float, and that LDPE, PP, and PS are recovered in both solutions due to their lower density, I have a question regarding the use of NaCl (1.20 g/cm^3). Since PET would settle in NaCl at the above density, I would like to suggest the authors to consider recovering the PET in a denser NaCl solution. If not, it would be good if explanation was provided for why this approach was not considered for the recovery of PET in NaCl.
2. Mention the purpose of the rapid cooling of the samples in line 126 and also provide the temperature.
3. Line 146: Briefly describe the calcination process and why it is used here?
