# Supplementary material for: Development of a new methodology for the determination of PET microplastics in sediment, based on microwave-assisted acid digestion
Source: PLoS One. 2024 Dec 17;19(12):e0314520. doi: 10.1371/journal.pone.0314520 (PMC11651601; doi:10.1371/journal.pone.0314520)
Supplement: Attachment — Submitted filename: Response to reviewers.docx [file pone.0314520.s012.docx]

PONE-D-24-22018

Development of a new methodology for the determination of PET microplastics in sediment, based on microwave-assisted acid digestion.

PLOS ONE

Dear Dr. Quiroz,

Thank you for submitting your manuscript to PLOS ONE. After careful consideration, we feel that it has merit but does not fully meet PLOS ONE’s publication criteria as it currently stands. Therefore, we invite you to submit a revised version of the manuscript that addresses the points raised during the review process.

Please submit your revised manuscript by Nov 20 2024 11:59PM. If you will need more time than this to complete your revisions, please reply to this message or contact the journal office at [plosone@plos.org](mailto:plosone@plos.org). When you're ready to submit your revision, log on to <https://www.editorialmanager.com/pone/> and select the 'Submissions Needing Revision' folder to locate your manuscript file.

Please include the following items when submitting your revised manuscript:

- A rebuttal letter that responds to each point raised by the academic editor and reviewer(s). You should upload this letter as a separate file labeled 'Response to Reviewers'.
- A marked-up copy of your manuscript that highlights changes made to the original version. You should upload this as a separate file labeled 'Revised Manuscript with Track Changes'.
- An unmarked version of your revised paper without tracked changes. You should upload this as a separate file labeled 'Manuscript'.

If you would like to make changes to your financial disclosure, please include your updated statement in your cover letter. Guidelines for resubmitting your figure files are available below the reviewer comments at the end of this letter.

If applicable, we recommend that you deposit your laboratory protocols in [protocols.io](http://protocols.io/) to enhance the reproducibility of your results. Protocols.io assigns your protocol its own identifier (DOI) so that it can be cited independently in the future. For instructions see: [https://journals.plos.org/plosone/s/submission-guidelines#loc-laboratory-protocols](https://c05y1x9s.r.us-east-2.awstrack.me/L0/https:%2F%2Fjournals.plos.org%2Fplosone%2Fs%2Fsubmission-guidelines%23loc-laboratory-protocols/1/010f019264930cce-7b883013-b87e-4116-b6bd-9f5d48793728-000000/vfwzKQZMTfiTI54RfjqRmrCzyAU=179). Additionally, PLOS ONE offers an option for publishing peer-reviewed Lab Protocol articles, which describe protocols hosted on [protocols.io](http://protocols.io/). Read more information on sharing protocols at [https://plos.org/protocols?utm_medium=editorial-email&utm_source=authorletters&utm_campaign=protocols](https://c05y1x9s.r.us-east-2.awstrack.me/L0/https:%2F%2Fplos.org%2Fprotocols%3Futm_medium=editorial-email%26utm_source=authorletters%26utm_campaign=protocols/1/010f019264930cce-7b883013-b87e-4116-b6bd-9f5d48793728-000000/D6LIZwBuMLTcWO9sP71ORJS3lw0=179).

We look forward to receiving your revised manuscript.

Kind regards,

Amitava Mukherjee, ME, Ph.D.
Academic Editor
PLOS ONE

Journal Requirements:

When submitting your revision, we need you to address these additional requirements.

1. Please ensure that your manuscript meets PLOS ONE's style requirements, including those for file naming. The PLOS ONE style templates can be found at

<https://journals.plos.org/plosone/s/file?id=wjVg/PLOSOne_formatting_sample_main_body.pdf> and

[https://journals.plos.org/plosone/s/file?id=ba62/PLOSOne_formatting_sample_title_authors_affiliations.pdf](https://c05y1x9s.r.us-east-2.awstrack.me/L0/https:%2F%2Fjournals.plos.org%2Fplosone%2Fs%2Ffile%3Fid=ba62%2FPLOSOne_formatting_sample_title_authors_affiliations.pdf/1/010f019264930cce-7b883013-b87e-4116-b6bd-9f5d48793728-000000/fLJY65fOGo_gZdRSrP3jWpeYiOs=179)

R: All check and changed according to PLOS ONE style requirements

2. In your Methods section, please provide additional information regarding the permits you obtained for the work. Please ensure you have included the full name of the authority that approved the field site access and, if no permits were required, a brief statement explaining why.

R: A statement was included at the end of method sections explaining that that, for this study, no special permits were required for sampling in the Loa River, as Chilean laws guarantee public access to river and sea shores.

3. We note that the grant information you provided in the ‘Funding Information’ and ‘Financial Disclosure’ sections do not match.

When you resubmit, please ensure that you provide the correct grant numbers for the awards you received for your study in the ‘Funding Information’ section.

R: Information was included in funding information section and erased from acknowledgment section

4. Thank you for stating the following financial disclosure:

“ANID-FONDECYT, Chile

Project 1230585.”

Please state what role the funders took in the study.  If the funders had no role, please state: "The funders had no role in study design, data collection and analysis, decision to publish, or preparation of the manuscript."

If this statement is not correct you must amend it as needed.

Please include this amended Role of Funder statement in your cover letter; we will change the online submission form on your behalf.

R: The funders had no role in study design, data collection and analysis, decision to publish, or preparation of the manuscript. This information was included in the cover letter and into the submission system

5. Thank you for stating the following in the Acknowledgments Section of your manuscript:

“The authors gratefully acknowledge financial support from ANID-FONDECYT, Chile

Project 1230585.”

We note that you have provided funding information that is not currently declared in your Funding Statement. However, funding information should not appear in the Acknowledgments section or other areas of your manuscript. We will only publish funding information present in the Funding Statement section of the online submission form.

Please remove any funding-related text from the manuscript and let us know how you would like to update your Funding Statement. Currently, your Funding Statement reads as follows:

“ANID-FONDECYT, Chile

Project 1230585.”

Please include your amended statements within your cover letter; we will change the online submission form on your behalf.

R: Funding information was erased from the manuscript and included in the cover letter and into the submission system

6. Please expand the acronym “ANID-FONDECYT” (as indicated in your financial disclosure) so that it states the name of your funders in full.

This information should be included in your cover letter; we will change the online submission form on your behalf.

R: Full Acronyms names ANID and Fondecyt were included in cover leter and in submission system

7. We note that your Data Availability Statement is currently as follows: [All relevant data are within the manuscript and its Supporting Information files.]

Please confirm at this time whether or not your submission contains all raw data required to replicate the results of your study. Authors must share the “minimal data set” for their submission. PLOS defines the minimal data set to consist of the data required to replicate all study findings reported in the article, as well as related metadata and methods (<https://journals.plos.org/plosone/s/data-availability#loc-minimal-data-set-definition>).

For example, authors should submit the following data:

- The values behind the means, standard deviations and other measures reported;

- The values used to build graphs;

- The points extracted from images for analysis.

Authors do not need to submit their entire data set if only a portion of the data was used in the reported study.

If your submission does not contain these data, please either upload them as Supporting Information files or deposit them to a stable, public repository and provide us with the relevant URLs, DOIs, or accession numbers. For a list of recommended repositories, please see <https://journals.plos.org/plosone/s/recommended-repositories>.

If there are ethical or legal restrictions on sharing a de-identified data set, please explain them in detail (e.g., data contain potentially sensitive information, data are owned by a third-party organization, etc.) and who has imposed them (e.g., an ethics committee). Please also provide contact information for a data access committee, ethics committee, or other institutional body to which data requests may be sent. If data are owned by a third party, please indicate how others may request data access.

R: All supplementary data was uploaded to one of the repositories recommended (OSF Home)

This include raw data of Figures 5, 7, 8, 9 and 12; For Table 1 and raw image used for MPs identification and quantification for Table 1 and Figures 8 and 12. Links were generated

https://osf.io/3rmqu/?view_only=f786d2c7739a40a2801ad39dbe72e407

All supported information was referenced in this new version.

Reviewers' comments:

Reviewer's Responses to Questions

**Comments to the Author**

**5. Is the article presented in an intelligible fashion and written in standard English?**

PLOS ONE does not copyedit accepted manuscripts, so the language in submitted articles must be clear, correct, and unambiguous. Any typographical or grammatical errors should be corrected at revision, so please highlight any specific errors that need correcting in the box below.

Reviewer #1: **No:** The overall flow of the manuscript must be improved.

R: Several grammatical errors were corrected, and the overall flow of the text was improved by incorporating connecting paragraphs and repositioning entire sections. We believe this new structure better follows the logical sequence of the validation process: first reproduction of staining Nile Red method, second the sample treatment validation; third, the color identification process; and finally, the application of the method to real samples.

6. Review Comments to the Author

The authors have formulated a way to recover PET particles from the sample by subjecting the

samples to differential conditions, including different temperature and excitation wavelength.

These conditions have successfully eliminated other interferences at each consequent stages

and resulted in the detection of PET MPs. I would like to suggest the following:

General comments: Applies to the entire manuscript.

1. Check the manuscript for grammatical and spelling errors. For example: In keywords

“Red Nyle” is not correct.

R: Red Nyle was corrected to Nye Red or NR in all manuscript

2. For all the statistical representation, mention the software used for the preparation of

graphs. Additionally, include a statistical analysis section for this in the methodology

head.

R: The "Statistical Analysis" section included details on the software used, the type of statistical tests, and the confidence level applied for each data analysis.

3. I suggest it would be appropriate if a pictorial representation or a flowchart is included

for methodology, including the effect of each step (such as elimination of chitin

fluorescence after digestion)

R: Fig. 2. Flowchart of the method was included according to reviewer advice. Representative pictures and the explicit purpose of each analytical step were included. This figure replace Figure 1 on this new version of the manuscript

4. The font consistency is lacking. For example: “Result and Discussions” and

“Conclusions” are in different forms; also check font in Table 1.

R: Font and size font was checked on all manuscript including head titles, table and figure labels “Vancouver font” was used in all manuscript and the size of the font and formatting were corrected according to Plos One “Manuscript body formatting guidelines”,

Hence, I recommend the authors to rewrite the manuscript considering all the above stated

suggestions and thus improving the overall flow of the manuscript.

Specific comments:

Major comments-

1. The addition of the possible real-life applications of this methodology would be

beneficial for the readers. Additionally, the methodology referred to in reference

number 9 has successfully recovered five polymer types. I suggest the authors to clearly

include a reason for their focus only on PET.

R: At the end of the discussion, an explanatory paragraph was added to clarify why this study focused on PET. Briefly, there are two main reasons. First, PET is one of the most frequently detected plastics in municipal waste. Locally, a published study has shown that many floating plastic containers have been found in Chilean rivers, including the Loa River, which is part of our study. Finally, our results indicate that this type of polymer was the most frequently observed in these samples. 3 references were included to support our choice. Finaly a monitoring was performed along sediments of Loa River in Chile. 4 sampling locations were included in this new version and results are presented in Figure 12.

2. It would be effective if the authors stated the reasons for each and every step as it will

provide great understanding for the readers.

R: This suggestion aligns with that made by the other reviewer. We recognize the importance of clarifying the purpose of each step in the methodology. For this reason, we have removed Figure 1 and replaced it with a pictorial flow diagram that includes the description and purpose of each methodological step. This is now Figure 2 in the revised manuscript.

3. Line 130- It would be good if the authors explained the use of only 254nm wavelength

filter and not the 365nm wavelength as mentioned in the reference paper.

R: The results of the Nile red-stained microplastics exposed to a 365 nm excitation wavelength were included, along with a discussion demonstrating that selective fluorescence was not achieved at this wavelength. This is now Figure 4 in the revised version of the manuscript.

Also state the efficiency of this method over already existing methods. Additionally, clarification of the possibility to recover other particles using different excitation wavelengths would be an added advantage. This information would be helpful since many researchers aim

to isolate all the potential plastic constituents from the environmental samples. Again,

I suggest the authors to clearly include a reason for their focus only on PET. For this

addition of microscopic images depicting the particles in bright field, the selected

fluorescence wavelength (254nm) and in other wavelengths, superimposed on each

other would substantiate the present study.

R: We greatly appreciate this reviewer’s comment, as it highlights the broader potential of the method we presented in the initial version of the manuscript. Indeed, this method has the potential to be extended to the identification and quantification of PE and PP. The reason we focused solely on PET is because this method was designed to be applied to the Loa River, which is primarily contaminated by PET plastic bottles, as described in a previous study that has been added to the introduction.

However, the identification of PE and PP still faces a selectivity challenge that we have not yet fully resolved. As shown in Figure 3, both PE and PP fluorescence colors are yellow with similar tones. Nevertheless, we agree with the reviewer that it is important to make readers aware of the real potential of this method.

Therefore, we have taken two actions: First, we used RGB color recognition through the artificial intelligence platform ChatGPT-4.0 to demonstrate two key points. Firstly, the method selectively detects PET. Secondly, while AI-based color recognition is possible for PE and PP, selectivity remains a challenge that must be addressed to fully unlock the method's potential.

For this reason, we have included Figure 9 to demonstrate the power of RGB color recognition for the three plastics (PET, PE, and PP) and Figure 10 to show the method’s selectivity for detecting PET, as well as the limitations regarding selectivity for PE and PP

Minor comments-

1. Considering that the density of PET is less than that of CaCl2, causing it to float, and

that LDPE, PP, and PS are recovered in both solutions due to their lower density, I have

a question regarding the use of NaCl (1.20 g/cm3). Since PET would settle in NaCl at

the above density, I would like to suggest the authors to consider recovering the PET

in a denser NaCl solution. If not, it would be good if explanation was provided for why

this approach was not considered for the recovery of PET in NaCl.

R: We acknowledge that presenting the flotation step results after the direct digestion method may give the impression that this step was not sufficiently optimized. However, the purpose of reproducing the flotation step is not to incorporate it into our procedure, but rather to demonstrate that it is unnecessary and that good recovery results can still be achieved by omitting this step and directly applying microwave digestion to the sample. To avoid further confusion, the flotation method results were presented first and discussed in this context, prior to introducing the recovery results from the direct digestion method.

2. Mention the purpose of the rapid cooling of the samples in line 126 and also provide

the temperature.

R: By apply this cycle of 50°C for staining and then 0°C for cooling have shown us that the particles exhibit more intense and longer-lasting fluorescence. We include the temperature and a proper discussion about this process.

3. Line 146: Briefly describe the calcination process and why it is used here?

R: Considering the heterogeneity in the distribution of microplastics in the environment, spike and recovery studies on real samples cannot be conducted using naturally contaminated samples because “native microplastics” cannot be subtracted mathematically. For this reason a calcination procedure was applied to a real sediment sample, to eliminate native MPs. A proper explanation was included at the end of “Trueness determination” method description according to reviewer advice.
